# Clinical relevance of serum omentin‐1 levels as a biomarker of prognosis in patients with acute cerebral infarction

**DOI:** 10.1002/brb3.1678

**Published:** 2020-06-01

**Authors:** Jingyi Yang, Yan Gao

**Affiliations:** ^1^ Department of Neurology Shengjing Hospital of China Medical University Shenyang China

**Keywords:** acute cerebral infarction, functional prognosis, modified rankin scale, omentin‐1

## Abstract

**Background and purpose:**

Previous studies have shown that adipocytokines are associated with atherosclerosis, diagnosis, and functional prognosis after ischemic stroke. However, few studies have investigated the relationship between omentin‐1 and atherosclerotic acute cerebral infarction (ACI).

**Methods:**

In this study, we investigated the association between serum omentin‐1 levels at admission and severity, infarction volume, and functional prognosis of patients 90 days after atherosclerotic ACI.

**Results:**

A total of 109 patients with atherosclerotic ACI were enrolled. Serum omentin‐1 levels at admission were lower in patients with ACI than those in healthy controls (47.18 ± 13.64 vs. 56.27 ± 34.44 ng/ml, *p* = .014). Serum omentin‐1 levels at admission were negatively correlated with severity of ACI (*r* = −.271, *p* = .004) and infarction volume (*r* = −.264, *p* = .006), respectively. Moreover, serum omentin‐1 levels were lower in the poor functional prognosis group than those in the good functional prognosis group in patients with large artery and small artery atherosclerotic ACI. In a logistic regression analysis, higher serum omentin‐1 level (>43.10 ng/ml) at admission was negatively associated with a poor functional prognosis 90 days after atherosclerotic ACI.

**Conclusions:**

Serum omentin‐1 levels at admission were significantly lower among patients with ACI. A higher plasma omentin‐1 level (>43.10 ng/ml) was negatively associated with poor functional prognosis 90 days after atherosclerotic ACI. Further studies are needed to investigate the pathophysiological mechanism of omentin‐1 in affecting attacks and prognosis of ACI as well as to confirm the value of plasma omentin‐1 level as a potential biomarker.

## Graphical abstract

Few studies have suggested a relationship between omentin‐1 and acute cerebral infarction (ACI), especially on atherosclerotic ACI. Serum omentin‐1 levels were significantly lower among ACI patients. Serum omentin‐1 levels at admission were negatively correlated with the severity and the infarction volume of ACI. Higher omentin‐1 plasma levels (>43.10 ng/ml) were negatively associated with poor functional outcome of patients at 90 days after atherosclerotic ACI.

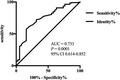

## 1 INTRODUCTION

Ischemic stroke is a clinical syndrome of local neurological defects that lasts longer than 24 hr. It is the first cause of disability in China. Studies have shown that more than 30% of patients with acute cerebral infarction (ACI) live with a permanent disability every year (Li et al., 2016), and atherosclerosis is the most common cause of ACI (Chiricozzi et al., 2016; Lobato et al., 2012). Adipose tissue is not only an energy storage organ, but also an important endocrine organ. The cytokines secreted by adipose tissue are called adipokines (Bloemer et al., 2018). Adipokines act on vascular smooth muscle cells, vascular endothelial cells, and macrophages to affect the state of the vascular wall and participate in the regulation of atherosclerosis (Ramirez et al., 2018). In recent years, researchers have found that the peripheral blood level of the adipokine omentin‐1 is significantly lower in patients with atherosclerosis and ischemic heart disease (Balli, Dogan, Dede, Sertoglu, & Keles, 2016; Kadoglou et al., 2015). Kadoglou et al. (2014) compared serum omentin‐1 levels in patients with severe atherosclerosis, mild atherosclerosis, and nonatherosclerosis and reported that the serum levels of omentin‐1 in patients with severe and mild atherosclerosis were lower than those in subjects without atherosclerosis. Xu, Zuo, Cao, Gao, and Ke (2018) determined that higher omentin‐1 levels were inversely related to carotid plaque instability and concluded that omentin‐1 may be a biomarker for predicting carotid plaque instability in patients with ACI. Omentin‐1 level can also be used as an independent predictor of carotid atherosclerosis (Shibata, Takahashi, et al., 2011), suggesting that omentin‐1 may be an atherosclerosis protective factor. Yue et al. reported that serum omentin‐1 concentrations are associated with the severity of cerebral infarction and decrease more significantly in patients with cardiogenic stroke than in those suffering from other kinds of stroke (Yue et al., 2018).

However, serum omentin‐1 levels are affected by many factors, such as heart failure and atrial fibrillation, changes in serum omentin‐1 levels in patients with cardiogenic stroke cannot be directly inferred to be associated with the stroke (Narumi et al., 2014; Tao et al., 2016). Moreover, as previous studies have concluded, serum omentin‐1 levels are significantly lower in patients with severe carotid atherosclerosis, and whether there is a different change in patients with small artery atherosclerosis is inconclusive.

In this study, we collected the data of patients with confirmed large and small arteriosclerotic ACI through different etiological types and detected the serum level of omentin‐1 at admission to clarify the relationship between serum omentin‐1 level and atherosclerotic ACI and its effect on the severity and infarction volume in patients with ACI. In addition, we determined the effect of serum omentin‐1 level on the prognosis of patients with arteriosclerotic ACI after the follow‐up.

## 2 METHODS

### 2.1 Study population

Patients diagnosed with ACI were enrolled in our study from the Department of Neurology, Sheng Jing Hospital of China Medical University between December 2016 and January 2018. Inclusion criteria were as follows: (a) acute onset, focal neurological deficit lasting >24 hr, admission to hospital for <72 hr; (b) results of diffusion‐weighted imaging (DWI) on magnetic resonance imaging (MRI) confirmed ACI; (c) subtypes of ACI were divided according to the Trial of Org 10172 in Acute Stroke Treatment (TOAST; Chen et al., 2012), patients consistent with large artery atherosclerosis (LAA) and small artery atherosclerosis (SAA) were enrolled; (d) agreed to be included in the study and accepted follow‐up. The exclusion criteria were as follows: (a) patients consistent with subtypes of ACI with cardioembolism, stroke of other determined etiology, and stroke of undetermined etiology; (b) other diseases that may affect omentin‐1 plasma levels, such as atrial fibrillation, rheumatic immune diseases, systemic and local inflammation, tumors, heart failure, severe heart disease, acute myocardial infarction, cirrhosis of the liver; and (c) failed to complete follow‐up. In addition, 109 healthy controls were recruited to form a control group.

The present study protocol was approved by the Medical Ethics Committee of the Sheng Jing Hospital of China Medical University. This study conformed to Ethical Guidelines for Medical and Health Research Involving Human Subjects endorsed by the Chinese government. This study did not interrupt any part of diagnosis or treatment, and all the clinical data would be proper preservation.

### 2.2 Assessment

The National Institutes of Health Stroke Scale (NIHSS) was used to assess the severity of neurological impairment at admission. Infarct volume was calculated on a follow‐up scan 48–72 hr after admission. The area of the DWI abnormality was outlined manually on the MR series. To calculate the total DWI lesion volume for each patient, the areas of DWI abnormalities were summed and multiplied by section thickness (mm) and intersection gap (mm). The average of the volume measurements was used as the volume of the ischemic lesion. The infarct volume of each patient was calculated three times, and the average was taken as the final infarct volume of the patient. The functional prognosis 90 days after ACI was assessed by the modified Rankin Scale (mRS). Patients with an mRS score of 0–2 were defined as the group with good functional prognosis, and those with an mRS score of 3–6 were defined as the group with poor functional prognosis.

### 2.3 Omentin‐1 measurements

Blood samples were obtained from patients between 6:00 a.m. and 7:00 a.m. after overnight fasting. Serum was centrifuged at 1509.3 *g* at 4°C for 15 min. The supernatant was stored in 1–2 ml aliquots at −80°C. The sera of patients with ACI were reexamined 7 days after admission, centrifuged, and preserved according to the above steps. Serum omentin‐1 levels were determined by enzyme‐linked immunosorbent assay. The human omentin‐1 kit was purchased from Shanghai Enzyme‐linked Biology Co. All experimental procedures were carried out in accordance with the manufacturer's instructions. The absorbance value was read at 450 nm, and the level of serum omentin‐1 was calculated from a standard curve.

### 2.4 Statistical analysis

Continuous variables are expressed as mean ± standard deviation. Mann–Whitney *U* test, Student's *t* test, or analysis of variance was used to compare continuous variables between groups. Categorical variables are expressed as a percentage, and the chi‐square test was used to compare the ratios between the groups. Prognostic accuracy was determined using receiver operating characteristic (ROC) curve analysis, and the critical point of the plasma omentin‐1 level was selected according to the Youden index. Potential confounding variables (gender and age) were corrected, and the effect of omentin‐1 on functional prognosis was assessed by binary logistic regression analysis. The results are shown as adjusted odds ratios (ORs) and 95% confidence intervals (CIs). All statistical analyses were performed using SPSS 22.0 software (SPSS Inc.). Statistically significant threshold levels were established at *p* < .05.

## 3 RESULTS

### 3.1 Sample characteristics

**FIGURE 1:**
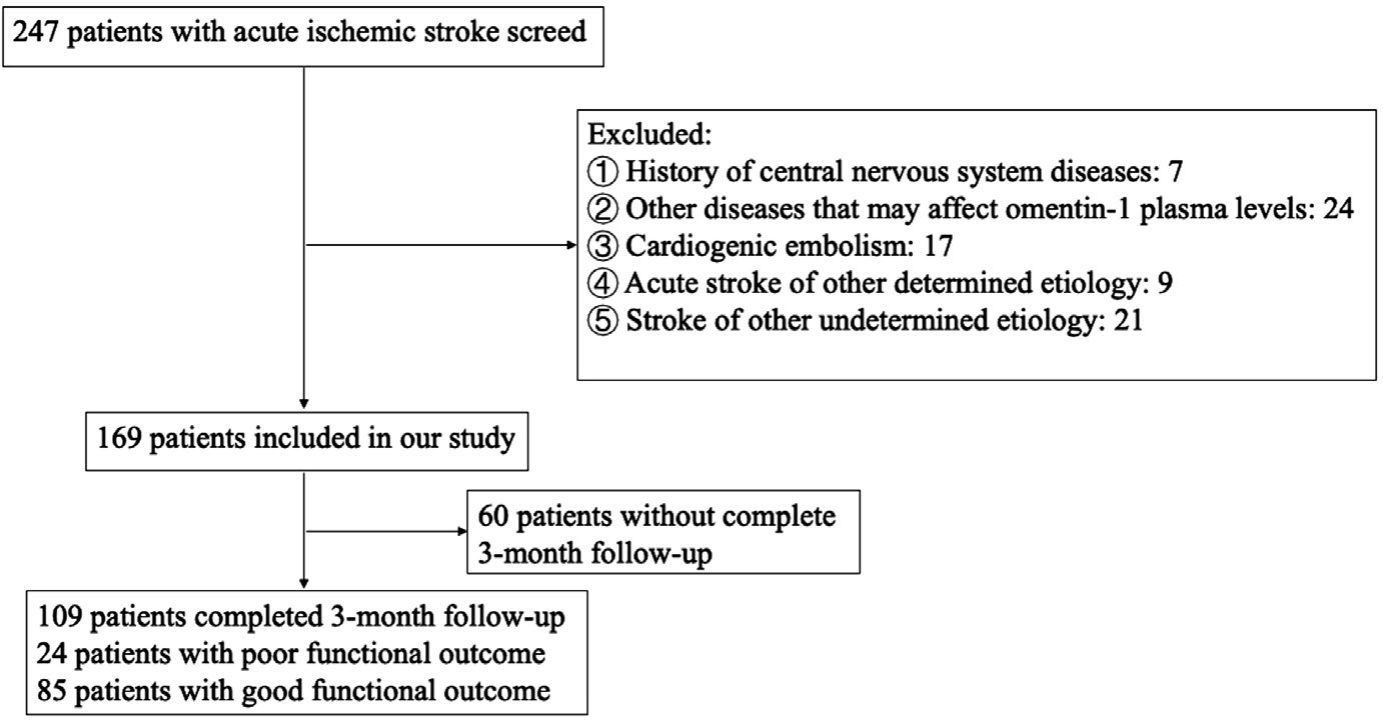
Study recruitment profile

**FIGURE 2:**
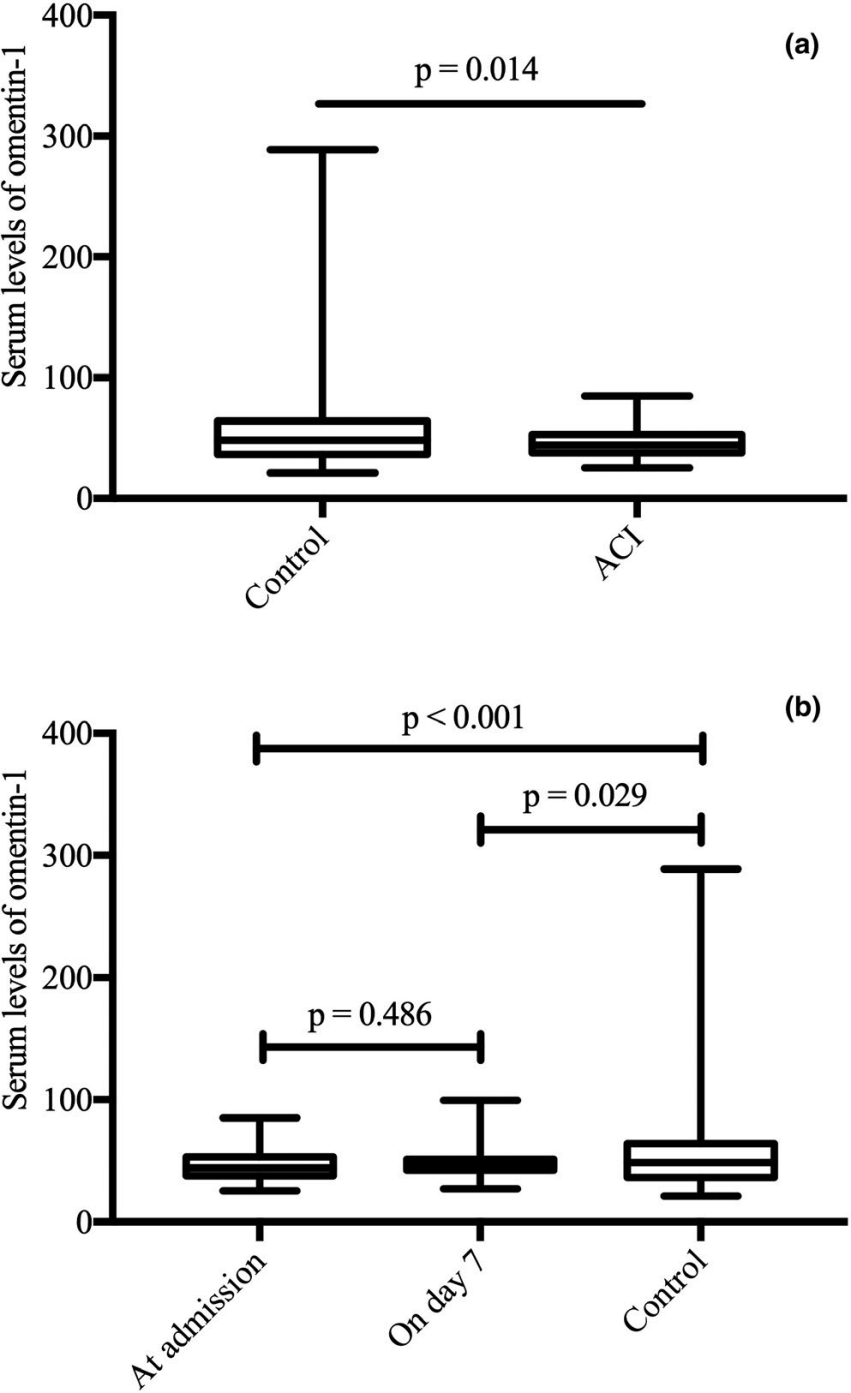
(a) Boxplots of omentin‐1 levels between acute cerebral infarction and control groups. (b) Boxplots of omentin‐1 levels between patients with acute cerebral infarction at admission and on day 7 and healthy controls

Totally, 247 patients with ACI underwent consecutive screening, and 169 patients met the inclusion criteria at baseline. At the 90‐day follow‐up, 109 patients were eventually enrolled in the study (Figure 1). Serum omentin‐1 levels at admission and on day 7 were significantly lower in the patients with ACI than those in the healthy controls (47.18 ± 13.64 vs. 56.27 ± 34.44 ng/ml, *p* = .014; Figure 2a,b). No differences were observed in the baseline characteristics, such as age or gender, between the patients with ACI and the healthy controls. The serum omentin‐1 levels at admission in the ACI groups (good and poor functional prognosis) were both significantly lower than those of the controls (Table 1).

**TABLE 1:** Clinical and demographic characteristics of the samples under study

Baseline characteristics	MRS 0–2 (*n* = 85)	MRS 3–6 (*n* = 24)	Healthy controls (*n* = 109)
Age, years, mean ± *SD*	62.44 ± 13.04	64.13 ± 9.68	62.98 ± 13.13
Gender, male, *n* (%)	54 (63.53)	14 (58.33)	66 (66.55)
History of hypertension, *n* (%)	51 (60.0)	17 (70.83)	
History of DM, *n* (%)	24 (28.23)	6 (25.0）	
History of coronary heart disease, *n* (%)	10 (11.76)	2 (8.33）	
History of smoking, *n* (%)	44 (51.76)	10 (41.67)	
History of alcohol, *n* (%)	31 (36.47)	8 (33.33)	
LAA, *n* (%)	30 (35.29)	11 (45.83)	
SAA, *n* (%)	55 (64.71)	13 (54.17)	
NIHSS score	2.09 ± 1.63	5.83 ± 3.24^a^	
Cerebral infarction volume (cm^3^)	1.30 ± 3.32	7.70 ± 10.21^a^	
Cholesterol (mmol/L)	4.68 ± 1.18	4.71 ± 1.08	
Triglycerides (mmol/L)	2.08 ± 1.99	1.47 ± 0.62	
LDL (mmol/L)	3.00 ± 0.98	2.96 ± 0.88	
HDL (mmol/L)	1.15 ± 0.60	1.19 ± 0.29	
HCY (mmol/L)	19.45 ± 17.59	15.46 ± 5.03	
Creatinine (μmol/L)	77.09 ± 64.64	90.60 ± 102.34	
BUN (mmol/L)	5.37 ± 1.71	5.37 ± 1.44	
Cystatin C mg/L	1.30 ± 0.84	1.12 ± 0.23	
HbA1c (%)	6.74 ± 1.70	6.13 ± 0.50	
Omentin‐1 at admission (ng/ml)	49.29 ± 13.45^b^	39.71 ± 11.76^a^, ^b^	56.27 ± 34.44

Note

Data are presented as number (percentage) or mean ± standard deviation, unless otherwise indicated.

Abbreviations: BUN, blood urea nitrogen; DM, diabetes mellitus, HbA1c, glycosylated hemoglobin; HCY, Homocysteine; HDL, high‐density lipoprotein cholesterol; LAA, large artery atherosclerosis; LDL, low‐density lipoprotein cholesterol; mRS, modified Rankin Scale; NIHSS, National Institutes of Health Stroke Scale; SAA, small artery atherosclerosis; *SD*, standard deviation.

^a^ *p* < .001 compared to MRS 0–2.

^b^ *p* < .001 compared to healthy controls.

### 3.2 Influence of serum omentin‐1 levels at admission on patients with ACI of the LAA/SAA subtype

**FIGURE 3:**
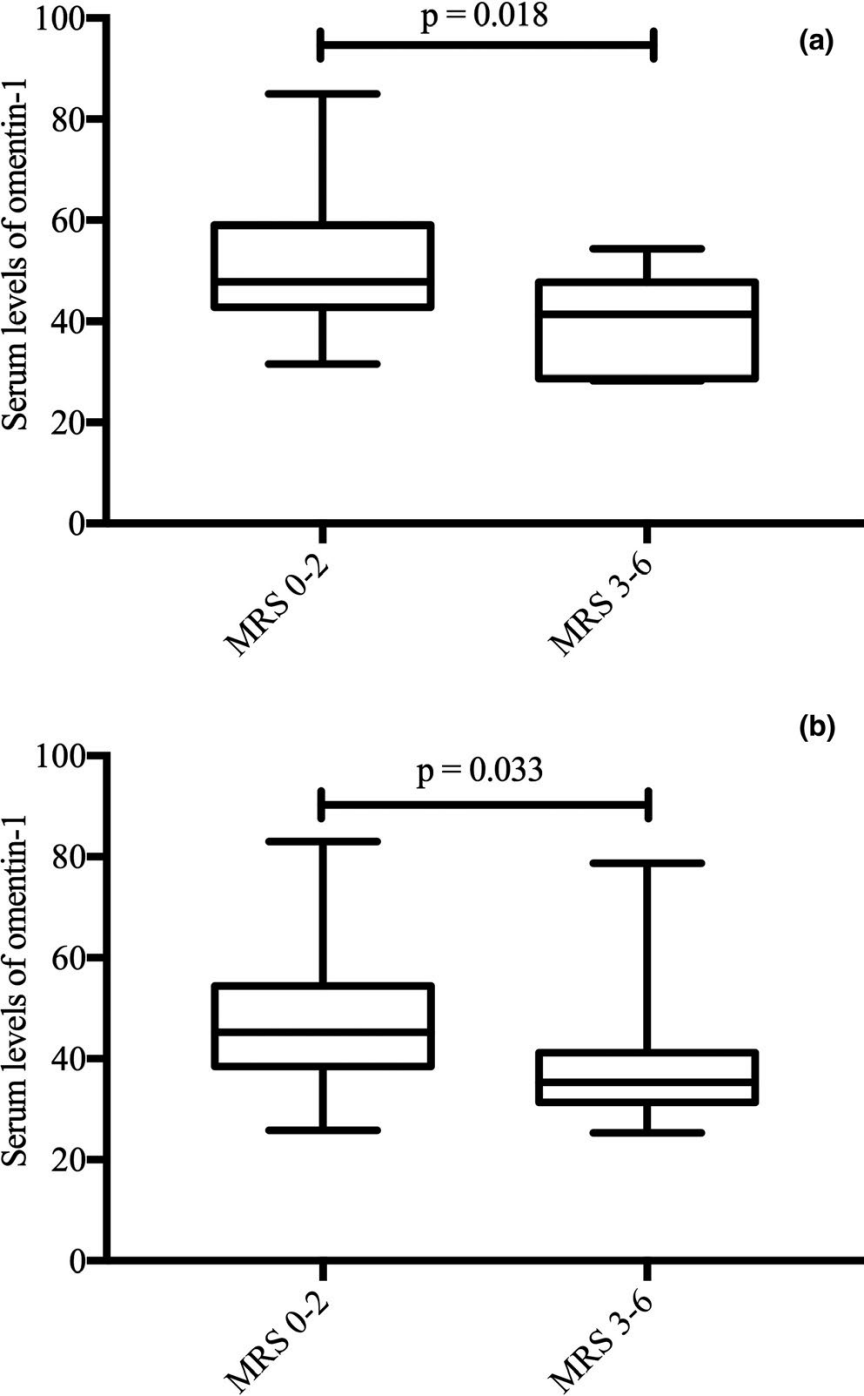
(a) Boxplots of omentin‐1 levels for patients with acute cerebral infarction and good and poor functional prognosis of the LAA subtype; (b) Boxplots of omentin‐1 levels for patients with acute cerebral infarction and good and poor functional prognosis of the SAA subtype. LAA, large artery atherosclerosis; SAA, small artery atherosclerosis

Through the TOAST classification, we finally divided the 109 patients with ACI into 41 cases of LAA (37.61%) and 68 cases of SAA (62.39%). According to the mRS score, 30 cases of ACI of the LAA subtype were placed in the good functional prognosis group and 11 cases were in the poor functional prognosis group. The LAA proportion in the poor functional prognosis group was higher than that in the good functional prognosis group (45.83% vs. 35.29%). Fifty‐five cases of ACI of the SAA subtype were in the good functional prognosis group, and 13 cases were in the poor functional prognosis group. The SAA proportion in the good functional prognosis group was higher than that in the poor functional prognosis group (64.71% vs. 54.17%). Additionally, we compared serum omentin‐1 levels at admission in both subtypes of ACI and found that omentin‐1 levels were significant lower in the poor functional prognosis group of the LAA (40.38 ± 9.57 vs. 50.86 ± 12.84, *p* = .018) and SAA (39.13 ± 13.71 vs. 48.43 ± 13.81, *p* = .033) subtypes than those of the good functional prognosis group, respectively (Figure 3a,b).

### 3.3 Factors associated with functional prognosis 90 days after ACI

The NIHSS score was significantly higher in the poor functional prognosis group than that in the good functional prognosis group (5.83 ± 3.24 vs. 2.09 ± 1.63, *p* < .001). Infarction volume was also larger in the poor functional prognosis group than that in the good functional prognosis group (7.70 ± 10.21 vs. 1.30 ± 3.32, *p* < .001). Serum omentin‐1 levels were lower in the poor functional prognosis group than that in the good functional prognosis group (39.71 ± 11.76 vs. 49.29 ± 13.45 ng/ml, *p* = .002; Table 1). No differences were observed in factors, such as age, gender, medical history, or biochemical test results.

**FIGURE 4:**
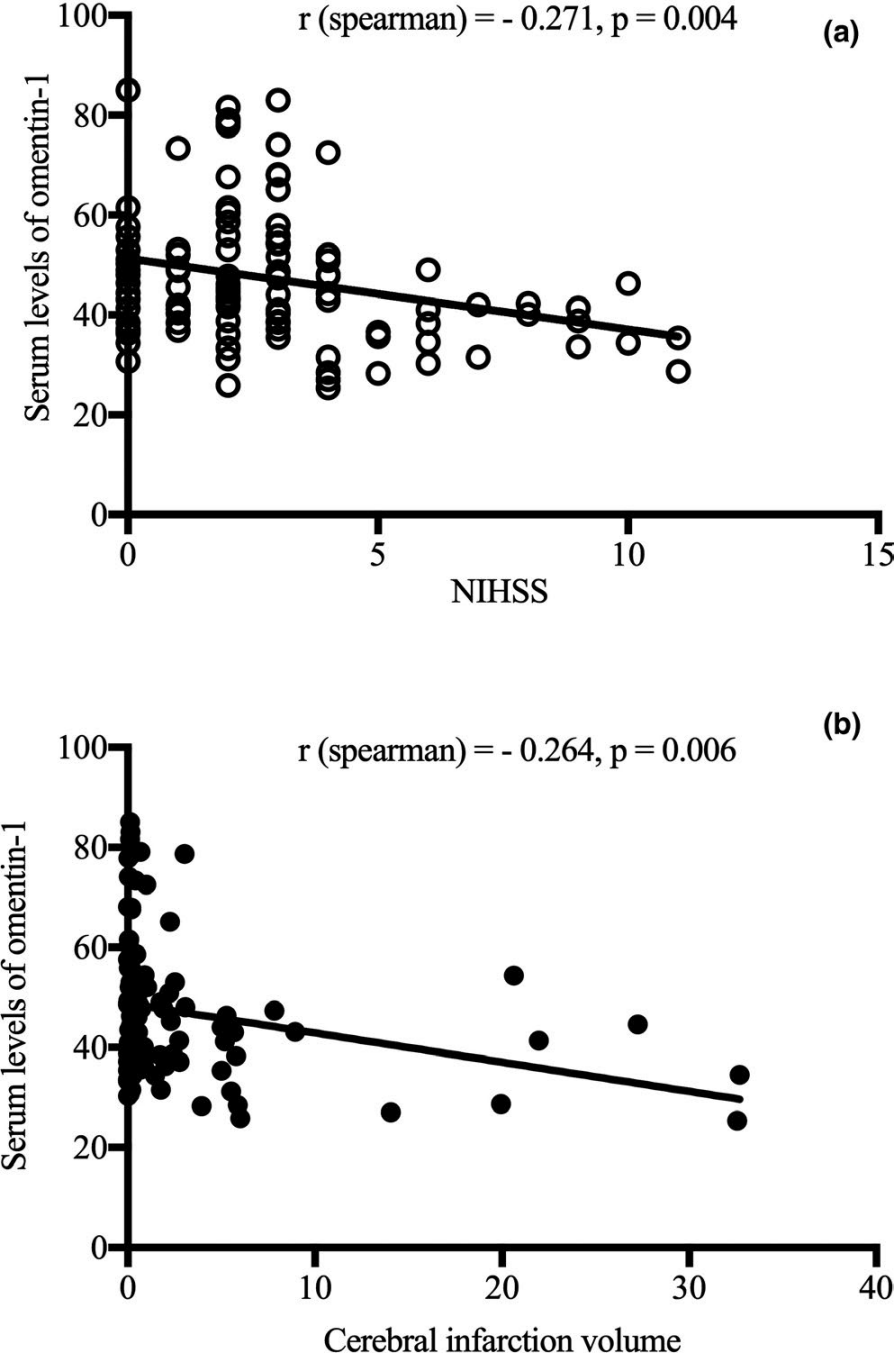
(a) Correlation between serum levels of omentin‐1 and the NIHSS score; (b) Correlation between serum levels of omentin‐1 and infarct volume. NIHSS, National Institutes of Health Stroke Scale

**FIGURE 5:**
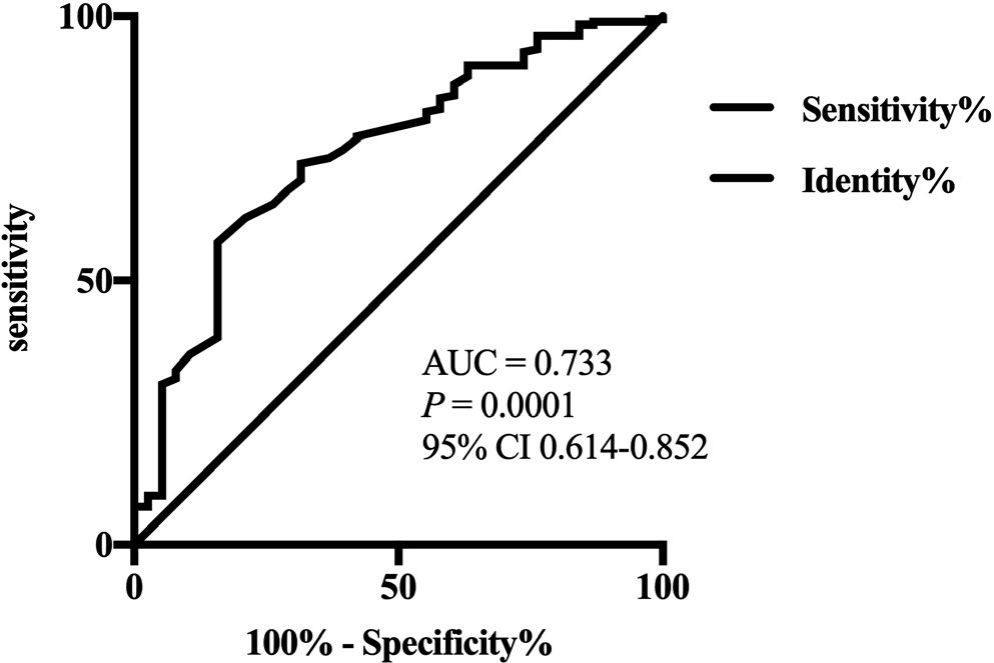
Receiver operator characteristic curve demonstrating sensitivity as a function of 1 − specificity for predicting the functional prognosis based on the plasma omentin‐1 level

In this study, serum omentin‐1 levels at admission were negatively correlated with the NIHSS score (*r* = −.271, *p* = .004; Figure 4a). In addition, a negative correlation was detected between serum omentin‐1 levels at admission and infarct volume (*r* = −.264, *p* = .006; Figure 4b). According to the ROC curve (Figure 5), the optimal cutoff serum omentin‐1 level as an indicator for an auxiliary diagnosis of 90‐day functional prognosis was 43.10 ng/ml, with a sensitivity of 76.5% and a specificity of 62.5% (AUC 0.733; 95% CI [0.614–0.852]; *p* = .001). Independent factors for predicting 90‐day functional prognosis after ACI were evaluated by binary logistic regression analysis (Table 2). The results showed that the NIHSS score (OR 1.840, 95% CI 1.336–2.532, *p* < .001) and cerebral infarction volume (OR 1.170, 95% CI 1.034–1.324, *p* = .012) were significantly correlated with poor functional prognosis. In addition, baseline plasma omentin‐1 levels above the cutoff (43.10 ng/ml) were negatively associated with poor functional prognosis among patients with ACI (OR 0.184, 95% CI 0.044–0.776, *p* = .021).

**TABLE 2:** Binary logistic regression of the clinical determinants of functional prognosis 90 days after stroke

Variables	OR (95% CI)	*p*‐value
NIHSS	1.840 (1.336–2.532)	<.001
Cerebral infarction volume	1.170 (1.034–1.324)	.012
Omentin‐1 at admission (>43.10 ng/ml)	0.184 (0.044–0.776)	.021

Abbreviation: NIHSS, National Institutes of Health Stroke Scale.

## 4 DISCUSSION

The main purpose of this study was to elucidate the changes in baseline serum omentin‐1 levels in patients with ACI. We also assessed the relationship between omentin‐1 levels and the severity of cerebral infarction, the volume of the cerebral infarction, and the impact on functional prognosis of patients 90 days after ACI. Our findings indicate that serum omentin‐1 levels decreased significantly in patients with ACI than those in the healthy controls, and no obvious change in our patients was detected on day 7. Serum omentin‐1 levels at admission were negatively correlated with the NIHSS score and infarction volume. In addition, a baseline serum omentin‐1 level >43.10 ng/ml was negatively associated with poor functional prognosis among patients with ACI. Therefore, we conclude that omentin‐1 has a protective effect on patients with ACI and may be a biomarker for predicting functional prognosis after ACI.

Recent studies have confirmed that omentin‐1 mediates cardiovascular protective effects. Shibata et al. confirmed that serum omentin‐1 levels were significantly lower in patients with coronary heart disease (CHD) than in healthy people, suggesting that serum omentin‐1 is closely related to the occurrence and development of CHD. Further studies have shown that the severity of coronary artery atherosclerosis is negatively correlated with serum omentin‐1 level (Matsuo et al., 2015; Shibata, Ouchi, et al., 2011). However, the relationship between omentin‐1 and carotid atherosclerosis remains controversial. Studies have shown that serum omentin‐1 levels are closely related to the degree of atherosclerosis and carotid intima‐media thickness (Yoo et al., 2013). It has been speculated that the mechanism is through inhibiting tumor necrosis factor‐mediated endothelial dysfunction in human endothelial cells (Shibata, Takahashi, et al., 2011). Serum omentin‐1 levels in an atherosclerosis group were significantly lower than those in a nonatherosclerosis group (Yamawaki et al., 2011), and the levels were negatively correlated with carotid intima‐media thickness (Liu, Wang, & Bu, 2011). Xu et al. concluded that higher omentin‐1 levels are negatively correlated with carotid plaque instability in patients with ACI, but not with moderate‐severe carotid stenosis or occlusion. Omentin‐1 may be a biomarker for predicting carotid plaque instability in patients with ACI (Xu, Zuo, Cao, et al., 2018).

The omentin‐1 level is associated with the severity and prognosis of ACI. Yue et al. studied the clinical data and plasma omentin‐1 levels of 239 patients with ACI and reported a negative relationship between omentin‐1 level and the risk of ACI (Yue et al., 2018). Thus, omentin‐1 may be a promising indicator of stroke and its severity. However, 84 patients with cardiogenic embolism accounted for 35.1% of all patients in their study. Previous studies have suggested that the level of omentin‐1 in patients with arrhythmias, particularly atrial fibrillation, is lower than that in healthy people. Also, 65 patients had other causes or unknown reasons, which may also affect the omentin‐1 level, so the conclusion cannot completely and objectively reflect the changes in omentin‐1 levels in patients with ACI. Xu et al. showed that higher baseline omentin‐1 levels are negatively correlated with poor functional prognosis in patients with ACI. Omentin‐1 may be a biomarker for predicting poor functional prognosis of patients with ACI (Xu, Zuo, Wang, Gao, & Ke, 2018). However, these studies did not exclude patients with cardiogenic stroke and other unknown causes.

In strict accordance with the TOAST classification, we collected the LAA and SAA subtypes of patients with ACI in the present study to elucidate the change in omentin‐1 levels in patients with atherosclerotic ACI and their impact on the prognosis of such patients. Our study found that the serum omentin‐1 levels of patients with ACI were lower than those of healthy controls at admission and on day 7 of admission, but there was no significant change in the omentin‐1 levels at two time points in the patients with ACI. Although most studies agree on the neuroprotective effect of omentin‐1, the mechanisms remain unclear. Current studies suggest that omentin‐1 may play a protective role through the following mechanisms: (a) Omentin‐1 may directly or indirectly inhibit inflammation, thereby reducing the formation and rupture of unstable plaques and prevent cerebrovascular events (Kazama, Usui, Okada, Hara, & Yamawaki, 2012). (b) Omentin‐1 inhibits oxidative stress in vascular smooth muscle cells, plays an anti‐atherosclerosis role, as well as inhibits oxidative stress in mitochondria, thereby reducing cytotoxicity and playing a neuroprotective role (Kazama, Okada, & Yamawaki, 2015). (c) Omentin‐1 promotes endothelial release of bioactive substances, regulates endothelial function, and improves vasomotor function (Yamawaki et al., 2011). Moreover, omentin‐1 inhibits alkaline phosphatase activity and osteocalcin production, thereby inhibiting the differentiation of vascular smooth muscle cells into osteoblast‐like cells to prevent vascular calcification (Kazama et al., 2015). Omentin‐1 also promotes vascular remodeling in the ischemic state to improve endothelial function, reduce infarct size, and reduce apoptosis (Kataoka et al., 2014). However, the exact protective mechanisms of omentin‐1 need further study.

A further logistic regression analysis showed that the NIHSS score and cerebral infarction volume were independent risk factors for a poor functional prognosis in patients with ACI. In this study, serum omentin‐1 levels were negatively correlated with the NIHSS score and cerebral infarction volume, which further confirmed the protective effect of omentin‐1 on patients with ACI. We further quantitatively analyzed the effect of serum omentin‐1 level on 90‐day functional prognosis of patients with ACI using a ROC curve. When serum omentin‐1 level was >43.10 ng/ml, the AUC was 0.733, sensitivity was 76.5%, and specificity was 62.5%, which revealed the best diagnostic value and was negatively correlated with the prognosis of ACI.

Some limitations of this study need to be considered. First, the study was conducted in a single center, so the sample size is limited. Second, the number of subjects in the poor functional prognosis group was small, which may result in inaccurate statistical results. Third, we recorded serum omentin‐1 levels of patients with ACI at admission and on day 7, but the dynamic changes of omentin‐1 levels were not monitored. Finally, few studies have researched the protective mechanism of omentin‐1 in ACI. Therefore, more cases and further mechanistic studies are needed.

## 5 CONCLUSIONS

Serum omentin‐1 levels in patients with ACI were significantly lower than those in healthy controls at admission and on day 7, but the level of omentin‐1 did not change at the two time points among the patients with ACI. Serum omentin‐1 level at admission was negatively correlated with the severity and infarction volume in patients with ACI. Higher plasma omentin‐1 levels (>43.10 ng/ml) were negatively associated with poor functional prognosis of patients 90 days after atherosclerotic ACI. Further studies are needed to investigate the pathophysiological mechanism of omentin‐1 in affecting attacks and the prognosis of ACI and to confirm the value of plasma omentin‐1 level as a potential biomarker.

## CONFLICT OF INTEREST

The authors declare that the research was conducted in the absence of any commercial or financial relationships that could be construed as a potential conflict of interest.

## AUTHOR CONTRIBUTION

Jingyi Yang collected clinical data, performed follow‐up of patients, completed statistical analysis, and wrote the manuscript. Yan Gao designed the study and contributed to editing the manuscript. All authors read and approved the final manuscript.

## Data Availability

The data of our study will be available via connecting with Dr. Yan Gao (corresponding author).
